# A Spanish Neuropsychological Battery Discriminates Between the Behavioral Variant of Frontotemporal Dementia and Primary Progressive Aphasia in a Colombian Sample

**DOI:** 10.3389/fneur.2021.656478

**Published:** 2021-07-05

**Authors:** Lina Velilla, Jonathan Hernández, Margarita Giraldo-Chica, Edmarie Guzmán-Vélez, Yakeel Quiroz, Francisco Lopera

**Affiliations:** ^1^Neuroscience Group of Antioquia, The University of Antioquia, Medellín, Colombia; ^2^Psychology Department, University Institution of Envigado, Envigado, Colombia; ^3^Harvard Medical School, Massachusetts General Hospital, Boston, MA, United States

**Keywords:** frontotemporal dementia, primary progressive aphasia, behavioral variant, neuropsychological tests, discriminant analyses

## Abstract

The differential diagnosis among the behavioral variant of frontotemporal dementia FTD (bvFTD) and the linguist one primary progressive aphasia (PPA) is challenging. Presentations of dementia type or variants dominated by personality change or aphasia are frequently misinterpreted as psychiatric illness, stroke, or other conditions. Therefore, it is important to identify cognitive tests that can distinguish the distinct FTD variants to reduce misdiagnosis and best tailor interventions. We aim to examine the discriminative capacity of the most frequently used cognitive tests in their Spanish version for the context of dementia evaluation as well as the qualitative aspects of the neuropsychological performance such as the frequency and type of errors, perseverations, and false positives that can best discriminate between bvFTD and PPA. We also described mood and behavioral profiles of participants with mild to moderate probable bvFTD and PPA. A total of 55 subjects were included in this cross-sectional study: 20 with PPA and 35 with bvFTD. All participants underwent standard dementia screening that included a medical history and physical examination, brain MRI, a semistructured caregiver interview, and neuropsychological testing. We found that bvFTD patients had worse performance in executive function tests, and the PPA presented with the lower performance in language tests and the global score of Mini-Mental State Examination (MMSE). After running the linear discriminant model, we found three functions of cognitive test and subtests combination and three functions made by the Montreal Cognitive Assessment (MoCA) language subtest and performance errors that predicted group belonging. Those functions were more capable to classify bvFTD cases rather than PPA. In conclusion, our study supports that the combination of an individual test of executive function and language, MoCA's subtest, and performance errors as well have good accuracy to discriminate between bvFTD and PPA.

## Introduction

Frontotemporal dementia (FTD) has been widely described as a syndrome that presents clinically by either behavioral/executive (BvFTD) or language dysfunction [i.e., primary progressive aphasia (PPA)]. These presentations are associated with prominent frontal or anterior temporal lobe degeneration (1) but with slightly different degeneration patterns and clinical profiles that merit distinct interventions. Yet, despite these differences and advances in molecular biomarkers and other diagnostic tools, differentiating between FTD variants themselves and other causes of dementia (e.g., Alzheimer's disease) remains a challenge. Presentations dominated by personality change or aphasia are readily misinterpreted as psychiatric illness, stroke, or other conditions (2).

In general, it has been described that, in PPA syndromes, most of the patients may debut with prominent anomia but with no frank semantic memory loss, and, additionally, those patients emerge with behavioral symptoms (3). This case evolution also involves only minor or mixed linguistic alterations and have a similar profile of behavioral change over time, mainly characterized by apathy (4), hindering differential diagnosis. For instance, the semantic variant of FTD (svFTD) is associated with behavioral disturbances that are similar in quality to those seen in bvFTD (5). Thus, the differential diagnosis among the PPA variants involves distinguishing among its variants themselves and discriminating among bvFTD and other causes of dementia (6). Therefore, it is important to identify cognitive tests that can distinguish the distinct FTD variants to reduce misdiagnosis and best tailor interventions. Furthermore, to the best of our knowledge, there are no studies yet that aimed to assess the discriminative capability of the Spanish version of widely used neuropsychological tests to evaluate cognitive changes between FTD and PPA, making it more needed to count on accurate neuropsychological data for the Latino population, where the access to sophisticated diagnostic technologies such PET-TAU and even to functional MRI (fMRI) is scarce.

The atrophy patterns associated with frontotemporal lobar degeneration (FTLD) have been found associated with family mutations in three genes, namely, chromosome 9 open-reading-frame 72 (C9ORF72), microtubule-associated protein tau (MAPT), and progranulin (GRN), and their clinical profiles are highly variable (7). For instance, GRN mutations are often characterized by prominent asymmetrical patterns of frontal, temporal, and parietal lobes atrophy, which are associated with behavioral changes, visuospatial deficit, and language disorders, resulting in most of the time on a clinical diagnosis of bvFTD or non-fluent variant PPA (nfvPPA) (8). Likewise, another study demonstrated that behavioral disturbances are common symptoms in sv-PPA; nearly 75% of the sv-PPA patients had at least one behavioral change at first presentation (4).

Previous studies have mostly focused on evaluating the capacity of screening and brief tools, as well as specific individual cognitive tests, to differentiate among FTD, AD, and healthy controls. However, although these tests shorten administration time, they also pose a challenge for effectively characterizing and differentiating dementia phenotypes (9).

Furthermore, qualitative aspects of the neuropsychological performance such as the frequency and type of errors, perseverations, false positives, and test's subitems can provide information about differential cognitive patterns of FTD variants. Prior research has found that specific errors in the neuropsychological test of memory such as false positives and intrusions are good markers of EA (10, 11). In FTD, there is a lack of research in this regard, although perseverations, discriminative errors, and paraphasias could have the potential of contributing to distinguish between bvFTD and PPA. Some previous studies support that hypothesis. For instance, previous research reported that phonological errors seem to be highly predictive of high amyloid burden in PPA (12). Similarly, another study found that both random and perseverative errors underlie the set-shifting deficits in the Wisconsin Sorting Cards Test (WSCT) test among patients with focal lesions to their lateral prefrontal cortex (13).

On the other hand, it has been proposed that the Montreal Cognitive Assessment (MoCA) subitems rather than the global scores can contribute to improving its discriminant capability. In a previous study, the authors reported that the MoCA subtests have not been extensively evaluated to explore its discriminative capacity, and they found that the subtest helped to discriminate among dementia, MCI, and healthy controls better than the MoCA global score alone (14). Another study aimed to explore the capability of the MoCA test subitems to examine cognitive deficits in FTD patients compared to single and longer measures. The authors found that all MoCA subitems, except the MoCA trials, strongly correlated with the corresponding full standard cognitive test and that the cognitive deficits related to FTD are better differentiated using MoCA subitems rather than the global score (15). Due to the lack of evidence showing a suitable capability to discriminate among dementias and FTD subtypes using the MoCA global scores and that some studies suggest that using the subitems could improve the discriminative capacity, we included the analysis of the MoCA subitems in our study.

For these reasons, we conducted a study that aimed to examine the discriminative capacity of a set of cognitive tests frequently used to evaluate patients with dementia and identifying subtest and qualitative aspects of the neuropsychological performance such as the frequency and type of errors, perseverations, and false positives that can best discriminate between bvFTD and PPA. We also assessed the cognitive, mood, and behavioral profiles of Colombian participants with mild to moderately probable bvFTD and PPA. The discriminant analysis is useful to indicate the most powerful combination of tests to distinguish between groups or to predict diagnostic group belonging regardless of the presence or the level of cognitive impairment. Since the discriminative capacity of the Spanish version of those test has not been assessed yet for the Latino population, this study will contribute to having better knowledge about the accuracy of this tool among Colombian patients. Moreover, we will have data about qualitative aspects of the neuropsychological evaluation, which can be useful for differential diagnosis in clinical settings and which have been little addressed in general.

## Materials and Methods

### Participants

A total of 55 subjects were included in this cross-sectional study: 20 with PPA and 35 with bvFTD. The inclusion criteria were the following: (a) the first clinical impression of a radiologist in fMRI was FTD and (b) the clinical evaluation by a neurologist suggested a differential diagnosis between bvFTD and PPA. The bvFTD group met the following criterion: (a) a clinical diagnosis of possible behavioral variant of FTD (supported by fMRI and Rascovsky et al. criteria) (15).

The PPA group fulfilled the following criterion: (a) clinical diagnosis of semantic variant and/or a non-fluent variant of PPA supported by fMRI and Gorno-Tempini et al. (16). Exclusion criteria for both groups were (a) significant motor disturbance that interfered with task performance and (b) patients with posterior cortical atrophy.

All subjects were recruited from the neuroscience group of Antioquia (GNA) data set (SISNE2), which include 30 years' worth of neurological, neuropsychological, genetic, and neuroimaging data from individuals who participate in research at the GNA. All participants underwent standard dementia screening that included a medical history and physical examination, brain MRI, a semistructured caregiver interview, and neuropsychological testing. The clinical diagnosis was established by consensus by a multidisciplinary team according to the fulfillment of inclusion/exclusion criteria. Informed consent was obtained from all subjects or their assigned surrogate decision-makers. The University of Antioquia institutional review boards for human research approved the study. The cognitive evaluation performed to evaluate diagnostic criteria fulfillment was different from the one used for this research's aim of assessing some cognitive test capability to discriminate between bvFTD and PPA. We used the Z-scores as our control data source. The Z-scores were made using Colombian normative data built with dementia cases compared to age-matched controls.

### Neuropsychological Background Testing

All cognitive tests were administered in the participants' primary language in Spanish by a trained neuropsychologist. Global cognitive performance was assessed with the Mini-Mental State Examination (MMSE) (17) and the MoCA (18). Memory performance was evaluated with the Colombian version of the Free and Cued Selective Reminding Test (FCSRT) (19). Visual–spatial skills and visual memory were evaluated with the Visual Memory Rey Complex Figure copy and immediate recall (17). We also evaluated subjective memory complaints with the subjective memory complaints patient/caregiver questionnaire (20). Executive function and behavioral symptoms were assessed with the Colombian version of the comprehensive cognitive battery Neuronorma, which includes the phonemic fluency test (p letter), the Stroop, and the Frontal Systems Behavior Scale (FRSB) (21). Other components of executive function such as flexibility and organized searching were assessed using the abbreviated and Colombian validation version of WSCT (17). Naming and semantic fluency were evaluated using the Neuronorma semantic fluency test (animals) and the Boston Naming Test (BNT) (21). The global/functional stage was explored with the Functional Assessment Staging Tool (FAST) (22) and the Global Deterioration Scale (GDS) (23).

### Statistical Analysis

Statistical analyses were performed using SPSS, version 25 (24). Group differences in demographics, disease severity scores, and neuropsychological, mood, and behavior measures were performed using Student's *t*-test for continuous variables and chi-square (χ^2^) for categorical variables, controlling for age and education using logistic regression with the diagnostic group as the dependent variable. The cognitive performance was standardized as Z-scores using previous normative data for the Colombian population. Errors, false positives, perseverations, and MoCA subitems were presented as median and standard deviation only, as there is no standardization yet for those measures among the Colombian population. Statistically significant variables on the bivariate analyses were then included in a linear discriminant function analysis (LDA) to determine how well-dementia subtypes can be distinguished based on the performance on cognitive tests. The LDA model is considered a robust technique that does not make the strong normality assumptions that multivariate ANOVA (MANOVA) does because the emphasis is on classification. Its robustness is not seriously affected if any of the assumptions are not met. A sample size of at least 20 observations in the smallest group is usually adequate to ensure the robustness of any inferential tests that may be made (25). Before running the model, we verified normality through visual strategies and statistical tests as Shapiro and Kolmogorov. Equality of covariance matrices was verified with the M. de Box test (26). We evaluated the assumption of no multicollinearity by calculating the Collinearity Statistics variance inflation factor (VIF). We found that our model fits very well all assumptions, and we consider it suitable to use the model that was performed using the group as a categorical independent variable and the cognitive features as independents. After checking eigenvalues and canonical correlations, we observed optimal values and coefficients. We assessed the differences between groups through Wilks' lambda value with its respective significance test chi-square and found significate differences between groups in each function.

## Results

### Demographic and Clinical Features of the Sample

Table 1 summarizes demographics, disease severity scores, and estimated age of dementia onset, which was estimated through clinical history and clinical interview. Significant differences between group were not found.

**Table 1:** Demographic and clinical data of the sample according to the diagnostic group.

	**bvFTD**	**PPA**	***P*-value**
N	35	20	
Age	66.5 (10.5)	66.9 (8.05)	0.902
Education (years)	12.1 (6.22)	15.5 (6.72)	0.064
Functional assessment staging of Alzheimer's disease (FAST)	4.61 (1.17)	4.26 (1.05)	0.296
Global dementia scale (GDS)	4.58 (1.06)	4.21 (0.98)	0.225
Family history of dementia (% yes)	29.40%	25.00%	0.735
Family history of dementia (% no)	50.00%	45.00%	
Family history of dementia (unknown)	20.60%	30.0%	
Sex (% female)	44.10%	55.00%	0.312

*Data of continuous variables are presented as mean its respective standard deviation (SD) with Student's t-test. Categorical data are presented as frequencies with percentages and chi^2^ tests*.

### Neuropsychological Profiles

**Figure 1:**
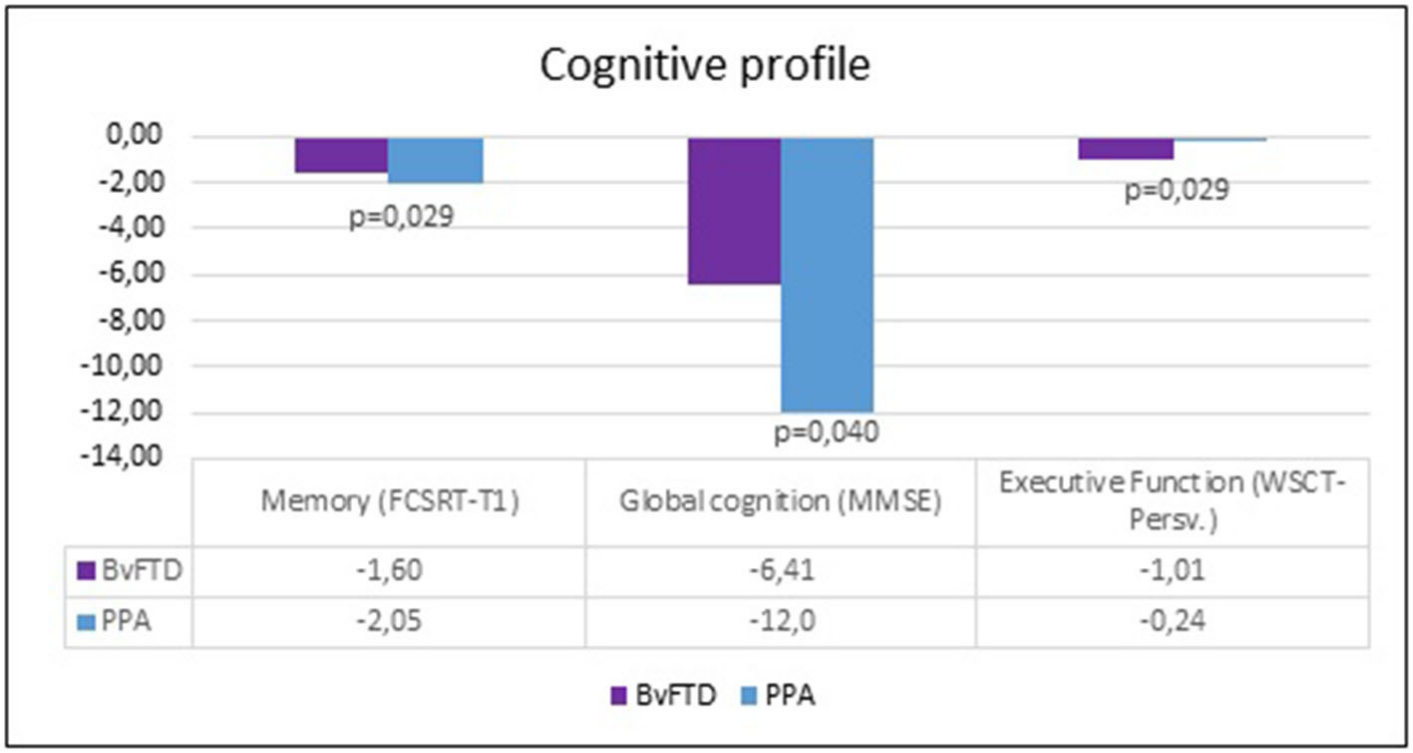
Global cognition, memory, and executive functions between FTD and PPA patients.

Table 2 summarizes the results of neuropsychological profiles of bvFTD and PPA patients. Differences were found in the MMSE, in the FCSRT (Trial 1 free recall) and WSCT (perseverative answers). Patients with bvFTD had a lower performance in executive function tests. Patients with PPA had a lower performance in the memory verbal span and MMMSE tests (Figure 1).

**Table 2:** Neuropsychological profiles of bvFTD and PPA patients.

	**bvFTD *n* = 35 Mean–(SD)**	**Qualitative range**	**PPA *n* = 20 Mean–(SD)**	**Qualitative range**	***P*-value**
**Global functioning**					
MoCA (Total)	−2.12 (0.84)	Extremely low	−2.08 (0.95)	Extremely low	0.891
MMSE	−6.41 (6.28)	Extremely low	−12.0 (9.40)	Extremely low	0.040^*^
**Memory**					
Trial 1 free recall	−1.60 (0.91)	Borderline	−2.05 (0.50)	Extremely low	0.029^*^
Total free recall	−2.23 (0.62)	Extremely low	−2.43 (0.48)	Extremely low	0.224
Total recall	−1.36 (1.86)	Borderline	−1.72 (1.67)	Borderline	0.496
Delayed free recall	−2.09 (0.59)	Borderline	−2.21 (0.65)	Borderline	0.526
Delayed total recall	−1.80 (1.41)	Borderline	−1.53 (1,91)	Borderline	0.59
**Executive function**					
Phonemic fluency P words (corrects)	−1.35 (1.18)	Borderline	−1.81 (1.03)	Borderline	0.138
Stroop (interference)	−1.69 (1.19)	Borderline	−1.87 (1.03)	Borderline	0.575
Wisconsin sorting cards (total corrects)	−0.78 (1.08)	Low average	−1.19 (1.24)	Borderline	0.065
Wisconsin sorting cards (perseveratives)	−1.01 (0.73)	Borderline	−0.24 (1.51)	Average	0.029^*^
Wisconsin sorting cards (categories)	−0.72 (1.00)	Low average	−0.87 (1.13)	Low average	0.632
Phonemic fluency (FAS)	−0.64 (1.30)	Average	−1.29 (1.03)	Borderline	0.052
**Language**					
Semantic fluency total (animals)	−2.28 (1.38)	Extremely low	−2.50 (1.45)	Extremely low	0.661
Naming (Total)	−1.38 (1.10)	Borderline	−1.68 (0.93)	Borderline	0.317
**Praxis**					
Praxis (CERAD-Col)	−1.62 (2.85)	Borderline	−1.96 (3.23)	Borderline	0.702
**Speed processing**					
Trials-A	2.01 (7.27)	High average	−0.03 (0.09)	Average	0.799

*Student's t-test was used to examine group differences. Presented values are Z-scores normalized for age and education (qualitative range of performance), using previously published normative data derived from Colombian samples (18–23). Qualitative range of performance was determined as such follows: ≤1 percentile rank = extremely low; 2–9 percentile rank = borderline; 9–24 percentile rank = low average; 25–74 percentile rank = average; 75–90 percentile rank = high average; 91–97 percentile rank = superior; ≥98 percentile rank = very superior*.

* *P < 0.05*.

Table 3 shows comparative performance in the MoCA subtest and errors, false positives, intrusions, and other pathological phenomena between the two groups. Results display significate differences between the MoCA language subtest where the PPA patients had the lower performance, in the number of incorrect p words for the phonemic fluency test with bvFTD presenting the higher number of errors, and in the number of descriptive errors for the naming test where the PPA group had the higher mean of pathological phenomena.

**Table 3:** Profile of errors, false positives, perseverations, and MOCA subtest of bvFTD and PPA patients.

	**BvFTD *n* = 35 Mean–(SD)**	**PPA *n* = 20 Mean–(SD)**	***P*-value**
**Global functioning**			
MoCa (total)	12.4 (7.23)	10.9 (7.47)	0.645
MoCa (visuospatial/executive)	2.21 (1.77)	2.05 (1.36)	0.074
MoCa (naming)	1.76 (1.13)	1.20 (1.19)	0.089
MoCa (attention)	2.65 (2.01)	2.10 (2.29)	0.374
MoCa (language)	1.15 (1.02)	0.45 (0.76)	0.011^*^
MoCa (abstraction)	0.62 (0.82)	0.45 (0.76)	0.450
MoCa (delayed recall)	0.53 (1.08)	0.70 (1.22)	0.608
MoCa (orientation)	3.24 (2.00)	3.70 (2.06)	0.423
**Memory**			
Intrusions	6.55 (6.25)	4.35 (4.87)	0.173
**Executive function**			
Phonemic fluency P words (incorrects)	1.09 (1.60)	0.29 (0.46)	0.009^*^
Phonemic fluency P words (perseverations)	0.47 (1.05)	0.14 (0,36)	0.103
**Language**			
Semantic fluency (animals perseverations)	0.41 (0.93)	0.10 (0.31)	0.072
Semantic fluency (animal intrusions)	0.06 (0.24)	0.05 (0.22)	0.862
Naming (descriptive errors)	1.03 (2.11)	3.85 (4.63)	0.013^*^
Naming (phonemic errors)	0.19 (0.40)	0.21 (0.54)	0.861
Naming (semantic errors)	3.63 (3.25)	2.74 (1.88)	0.222

*Data are presented as mean and standard deviation (SD). Student's t-test was used to examine group differences*.

* *P < 0.05*.

### Behavioral and Mood Profiles

Table 4 summarizes the mean and standard deviation of group performance in the behavior and mood tests. There were no significant differences in any variable.

**Table 4:** Behavioral and mood profiles of bvFTD and PPA patients.

	**BvFTD *n* = 35 Mean–(SD)**		**PPA *n* = 20 Mean–(SD)**		***P*-value**
**Behavior**					
FRSB behavioral change total	2.30 (2.00)		2.00 (1.61)		0.541
**Mood**	**Mean–(SD)**	**Qualitative range**	**Mean–(SD)**	**Qualitative range**	
Geriatric depression scale (GDS)	4.57 (6.56)	(No depression)	6.00 (15.3)	(Mild depression)	0.705
Zung depression scale	31.9 (13.9)	(No depression)	30.0 (10.7)	(No depression)	0.689

*Data are presented as mean and standard deviation (SD). Student's t-test was used to examine group differences. FRSB scores were Z-scores normalized for age and education. Scores for mood are additionally qualitative ranged using cut-points accordingly to previously published normative data*.

### The Discriminant Capacity of the Cognitive Battery Tests, Subtest and Errors, Intrusions, False Positives, and Other Pathological Phenomena

**Figure 2:**
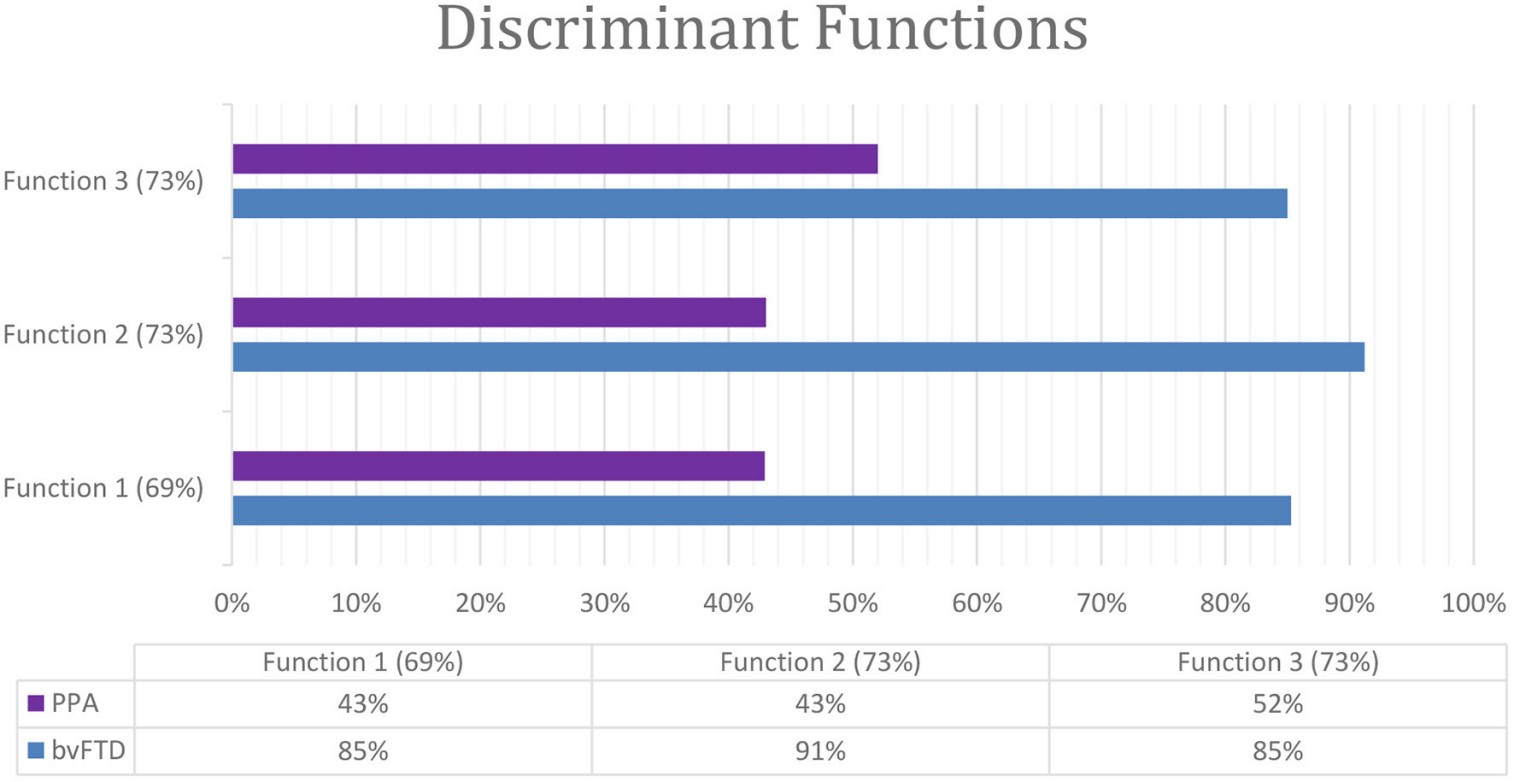
Classification of dementia by the discriminant functions as a combination of cognitive test.

Linear discriminant analyses were performed to identify the test combination with the highest capacity to differentiate between bvFTD and PPA. The discriminant function analysis model included the dementia subtypes as the grouping variable and the significant cognitive tests as discriminating variables. Three significant functions were found in the linear model, and they were able to classify correctly over 69% of cases. Function 1 was composed of MMSE and the FCSRT (trial 1 free recall) and classified correctly 69% of cases. This function classified correctly 85% bvFTD cases and 43% PPA cases (Wilks' ƛ = 0.841, chi^2^ = 6,252, *P* < 0.044). Function 2 was made of MMSE and WSCT (perseveratives) and classified correctly 73% of cases. This function classified correctly 91% bvFTD cases and 43% PPA cases (Wilks' ƛ = 0.762, chi^2^ = 9,773, *P* < 0.008). Function 3 combined WSCT (perseveratives) and FCSRT (trial 1 free recall) and classified correctly 73% of cases. This function classified correctly 85% bvFTD cases and 52% PPA cases (Wilks' ƛ = 0.802, chi^2^ = 7,935, *P* < 0.019) (Figure 2).

### The Discriminant Capacity of Subtest and Errors, Intrusions, False Positives, and Other Pathological Phenomena

**Figure 3:**
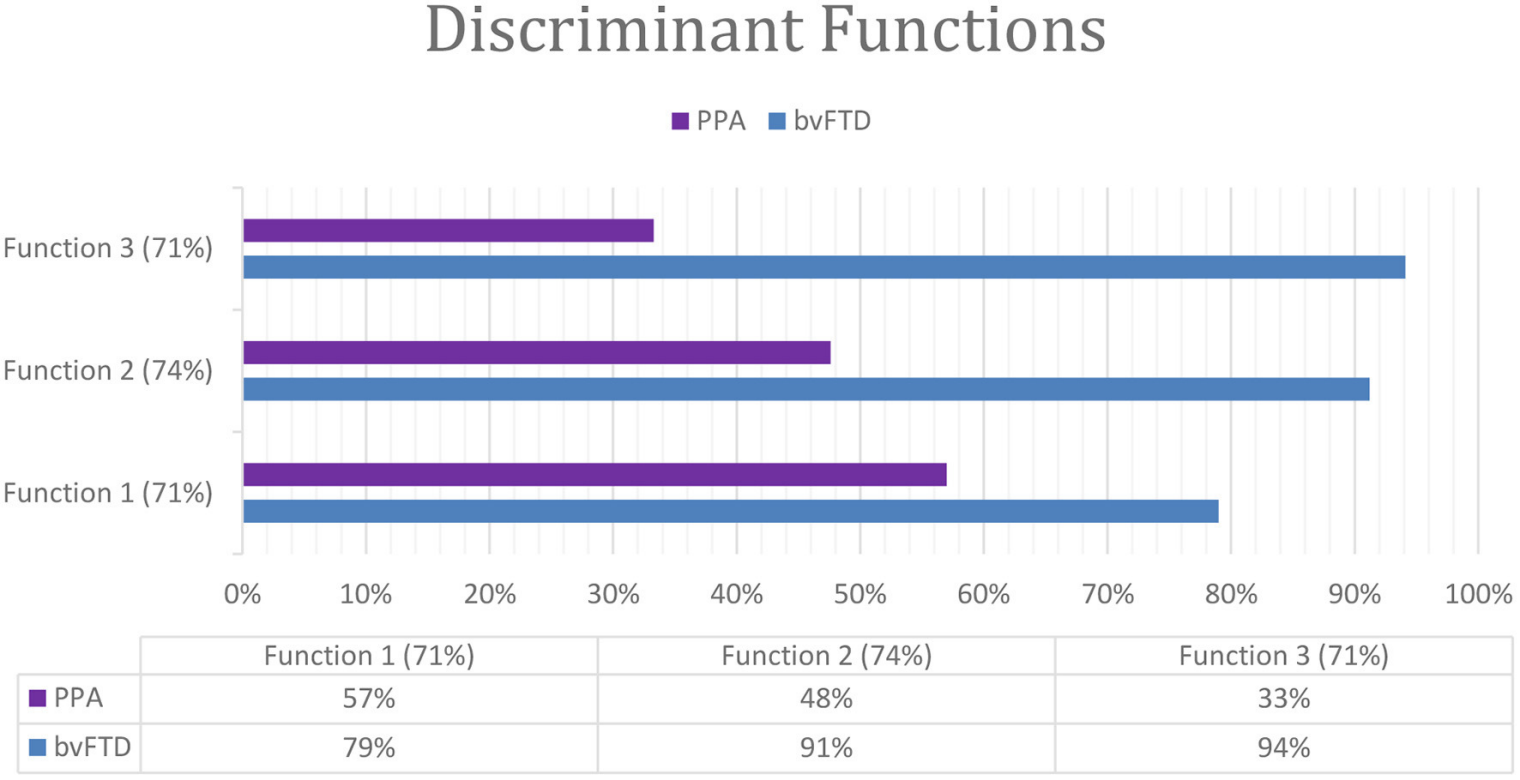
Classification of dementia by the discriminant functions (subtest and errors).

We included the discriminative analyses using a combination of MoCA subtest and errors. We found three significate combinations able to classify correctly up to 74% of cases. Function 1 was composed of MoCA (language subtest) and the mean of incorrect p words (phonemic fluency) and classified correctly 71% cases. This function classified correctly 79% bvFTD cases and 57% PPA cases (Wilks' ƛ = 0.826, chi^2^ = 9,755, *P* < 0.008). Function 2 was composed of MoCA (language subtest) and the mean of descriptive errors for the naming test and classified correctly 74% cases. This function classified correctly 91% bvFTD cases and 48% PPA cases (Wilks' ƛ = 0.791, chi^2^ = 11,033, *P* < 0.004). Function 3 was composed of incorrect p words (phonemic fluency) and descriptive errors for the naming test and classified correctly 71% cases. This function classified correctly 94% bvFTD cases and 33% PPA cases (Wilks' ƛ = 0.802, chi^2^ = 10,606, *P* < 0.005) (Figure 3).

## Discussion

In this cross-sectional study, we assessed the capability to discriminate between bvFTD and PPA of the Spanish version of a set of cognitive tests set widely used in dementia diagnosis in a sample of Colombian patients. The battery tests included measures of memory, executive function, language, praxis, and global functioning. We also described mood, subjective cognitive decline, and behavioral changes between the two groups.

We introduced the cognitive variables that significantly discriminated those with bvFTD from PPA into a linear discriminant function model to establish the discriminant functions that better contribute to predicting whether a patient belongs to the bvFTD or PPA group.

As a group, bvFTD patients had lower education compared to PPA patients presenting significant differences for that variable. There were no significant differences in other demographic and clinical background variables, although it is noticeable that a pattern of higher frequency of family history of dementia among the bvFTD patients. The clinical dementia stage mean was four (4) measured by the FAST scale (22) with no differences between groups. The participants were evaluated while presenting mild to moderate dementia. Assessing the participants in the course of those stages is timely to have relevant data to be extrapolated in the clinical practice mostly in Colombia where patients usually access to neurological consultation when the dementia stage is advanced; accordingly, it is considered even more pertinent to have data on this population in the intermediate and advanced stages of the disease. The bvFTD group exhibited better performance at the MMSE and the verbal memory span. They also exhibited more deficits in executive function. We analyzed intragroup differences in MoCA subtests, errors, perseverations, false positives, and other pathological phenomena. We found that the PPA patients presented a lower performance in the MoCA language subtest and significatively a higher number of descriptive errors in the Boston naming test. On the other hand, the bvFTD group presented a higher number of errors at the executive function test phonemic fluency (p words). In summary, we found that bvFTD patients had worse performance in executive function tests, and the PPA presented with the lower performance in language tests and the global score of MMSE. Those results are the same with that of Osher et al. (27) where PPA patients presented with a lower decline in the MMSE compared to bvFTD patients who correlated strongly with the decline in MMSE and the Activities of Daily Living Questionnaire (ADLQ) overtime.

After running the linear discriminant model, we found three functions of cognitive test and subtests combination and three functions made by the MoCA language subtest and performance errors that predicted group belonging with a global discriminant capacity of 74 and 71%. Our results are the same as the findings of Kramer et al. (28). They found that the combination of some performance errors and cognitive test, such as Boston Naming, modified Rey recall, CVLT-SF recall, category fluency, and executive errors produced two canonical functions able to discriminate between bvFTD, a linguistic variant of FTD and AD with a global discriminant capacity of 87.7% (28).

Other previous studies included MoCA subtests like Milani et al. (29) who found in a large sample study that, among Hispanics, the MoCA subtests had higher discrimination and more diagnostic utility (29). Similarly to our study, another study analyzed the capability of subtest from a cognitive battery to contribute to differential diagnosis in dementia. The authors reported that the subtests provide efficient and valid measures of neurocognition that are key for differential diagnosis (30).

Studies of clinicopathological correlation have shown that the most common underlying pathology in PPA is bvFTD (31, 32). Hence a high heterogeneity has been reported among the symptoms and clinical variants. In most cases, primary pathology of PPA and bvFTD is associated with neuropathological changes including tau or ubiquitin/TDP-43-positive inclusions; still, atypical Alzheimer's disease (AD) may also occur (33).

Current evidence demonstrates the existence of a consistent heterogeneity in the cognitive presentation of bvFTD syndrome (34–37) and, a large overlap between early bvFTD and other neurodegenerative diseases (including AD) (38). Similarly, PPA presentation includes an important range of heterogeneity, making it difficult to differentiate clearly between language affection as the hallmark described in PPA and other cognitive impairments that may co-occur, such as learning and memory, executive, and visuospatial functions (39).

A previous research has found that executive dysfunction is not necessarily the main trait of FTD and may even be absent on formal neuropsychological evaluation, particularly when examining total quantitative scores rather than using a qualitative approach to examine errors (40). Our findings are highly aligned with this study. We found that errors as perseverations were able to show significant differences among groups; those errors are attributable to executive function failures and altered linguistic performance. Similarly, other previous research aimed to analyze qualitative differences between FTD and AD. They reported that concrete thought, perseveration, confabulation, and poor organization, which disrupted performance across the range of neuropsychological tests, contributed to distinguish between both diagnostic entities. The authors explained that quantitative scores alone are limited in discriminative capacity, but performance characteristics and error types enhance the capacity to differentially diagnose, and qualitative information should be included in neuropsychological research and clinical assessments (41).

In this study, the combination of language and executive function subtest presented the highest discriminant capacity to discriminate between bvFTD and PPA cases. Previous research found similar results after analyzing cognitive subtests to discriminate among dementia groups. For instance, a research study found that the subtest of naming and executive functions has the most capability to distinguish between FTD and other dementias (42). Similarly, a group of authors performed a linear discrimination function for distinguishing between AD and FTD in the earlier dementia stages. They found that the combination of executive function subtest plus behavioral questions accurately classified 97% of individuals (43). Other research used the discriminant analysis model to differentiate among PPA variants; they found that linguistic subtests were able to classify correctly between 78 and 80% (44).

Different studies have studied the capacity of the cognitive tests to differentiate among different types of dementia, mainly between FTD and AD or within PPA variants. Nonetheless, the test discriminative capacity to distinguish globally between vbFTD and PPA cases has been scarcely addressed. Even more, it has not had been tested until now for the Spanish version of the cognitive tests assessed or among the Latino and, specifically, Colombian population. The distinction between FTD and PPA is relevant to clinical practice and reliable assessment of language, memory, and executive deficits, and it is paramount to distinguish the two conditions because, currently, it is well-known that PPA cases often start and/or develop with behavioral changes (45). Hence, the correct characterization of the cognitive deficits happening in the development of those conditions is key for diagnostic accuracy.

In conclusion, our study supports that the combination of an individual test of executive function and language, MoCA's subtest, and performance errors as well have good accuracy to discriminate between bvFTD and PPA. In our models, the tests were more accurate in classifying bvFTD cases. Those results point out that the neuropsychological examination of FTD and PPA must include linguistic and executive function tests together and that the qualitative analyses of neuropsychological results during the routine neuropsychological evaluation should include the performance errors and subtest to improve clinical reliability in distinguishing bvFTD from PPA. Our results also brought out the need to standardize MoCA subtests and the performance mistakes in the cognitive tests to improve the predictive capacity of neuropsychological evaluation to distinguish among FTD variants.

### Limitations

This study encompasses as a limitation the lack of genetic confirmation for FTLD mutations. Further research is needed to correlate the genetic confirmatory status, clinical diagnosis, and the capacity of the cognitive tests to discriminate among FTLD variants. Additionally, it is necessary to include in further research the PPA variants to assess the individual test capacity to differentiate among them. Since the sample size of this study is limited, we consider it as a pilot study to be continued, which should include a larger number of patients.

## Data Availability Statement

The raw data supporting the conclusions of this article will be made available by the authors, without undue reservation.

## Ethics Statement

The studies involving human participants were reviewed and approved by Institutional review board research headquarters Antioquia University (CBE-SIU). The patients/participants provided their written informed consent to participate in this study.

## Author Contributions

LV contributed to study design, database preparation, statistical analysis, clinical evaluation, and manuscript writing. JH contributed to study design and statistical analysis. MG-C contributed to clinical evaluation. EG-V contributed to data interpretation and the manuscript edition. YQ and FL contributed equally to study direction, manuscript revision, and mentoring. All authors contributed to the article and approved the submitted version.

## Conflict of Interest

The authors declare that the research was conducted in the absence of any commercial or financial relationships that could be construed as a potential conflict of interest.
